# The Cannabinoid Ligand Arachidonyl-2′-Chloroethylamide (ACEA) Ameliorates Depressive and Overactive Bladder Symptoms in a Corticosterone-Induced Female Wistar Rat Model

**DOI:** 10.3390/ijms24043820

**Published:** 2023-02-14

**Authors:** Łukasz Zapała, Grzegorz Niemczyk, Piotr Zapała, Artur Wdowiak, Iwona Bojar, Tomasz Kluz, Aleksandra Szopa, Anna Serefko, Piotr Radziszewski, Andrzej Wróbel

**Affiliations:** 1 Clinic of General, Oncological and Functional Urology, Medical University of Warsaw, Lindleya 4, 02-005 Warsaw, Poland; 2 Chair of Obstetrics and Gynecology, Faculty of Health Sciences, Medical University of Lublin, 4-6 Staszica St., 20-081 Lublin, Poland; 3 Department of Women’s Health, Institute of Rural Health in Lublin, Ul. Jaczewskiego 2, 20-090 Lublin, Poland; 4 Department of Gynecology, Gynecology Oncology and Obstetrics, Institute of Medical Sciences, Medical College of Rzeszow University, 16c Rejtana Street, 35-959 Rzeszow, Poland; 5 Department of Clinical Pharmacy and Pharmaceutical Care, Faculty of Pharmacy, Medical University of Lublin, 1 Chodźki Street, 20-093 Lublin, Poland; 6 Second Department of Gynecology, Medical University of Lublin, Jaczewskiego 8, 20-090 Lublin, Poland

**Keywords:** cannabinoids, cannabinoid receptors, overactive bladder, ACEA

## Abstract

There is growing need to increase the knowledge on the cannabinoid ligands in the treatment of overactive bladder. Among potential candidates, arachidonyl-2′-chloroethylamide (ACEA), a selective cannabinoid CB1 receptor agonist is proposed. The aim of this paper was to determine if ACEA, a selective cannabinoid CB1 receptor agonist, could reverse the effects of corticosterone (CORT), characteristic of depressive and bladder overactivity potential. The animals (48 female rats) were divided into four groups: I—control, II—received CORT, III—received ACEA, and IV—received the combination of CORT and ACEA. The conscious cystometry, forced swim test (FST), and locomotor activity measurements were performed 3 days after the last dose of ACEA, followed by ELISA measurements. In group IV, ACEA restored urodynamic parameters that were altered by CORT. CORT prolonged the immobility time in FST and the values were lowered by ACEA. ACEA normalized the expression of c-Fos in all the analyzed central micturition centers (group IV vs. group II). ACEA restored the CORT-induced changes in the biomarkers in urine (BDNF, NGF), bladder detrusor (VAChT, Rho kinase), bladder urothelium (CGRP, ATP, CRF, OCT-3, TRPV1), and hippocampus (TNF-α, IL-1β and Il-6, CRF, IL-10, BDNF, NGF). In conclusion, ACEA was proven to reverse CORT-induced changes in both cystometric and biochemical parameters that are determinants of OAB/depression, which represents an example of an existing link between OAB and depression via cannabinoid receptors.

## 1. Introduction

The syndrome of overactive bladder (OAB) consists of a group of lower urinary tracts symptoms, including increased voiding frequency, urgency and/or urge urinary incontinence, and nocturia, and may originate from urodynamic detrusor overactivity (DO) [1]. As a consequence, a direct link exists between DO and incontinence and storage symptoms, which in turn are thought to account for the symptoms which most severely affecting patients’ quality of life. Thus, the rush continues as far as the establishment of an adequate, efficient, and safe medical treatment of OAB [2].

Recently, there has been growing interest in cannabinoid receptors (CB), as novel agents for the treatment of lower urinary tract disorders that were already investigated in animal models [3,4]. Two major receptors, i.e., CB1 and CB2, were reported to be involved in these actions, the expression of which was found in central and peripheral nervous systems for the first type, and in glial/immune cells for the latter one, both in animals [4,5] and humans [6]. It is thought that stimulation of cannabinoid activity decreases Aδ- and C-fiber hyperactivity in the bladder neurons and, in turn, inhibits bladder overactivity, making these receptor ligands true candidates for targeting lower urinary tract symptoms [7]. Furthermore, the potential of the cannabinoid receptor ligands to influence bladder contractility was observed [8,9]. Nevertheless, the role of cannabinoid receptors in bladder physiology and pathophysiology is far from being clear, and only a greater understanding of the function of the peripheral CB1 and CB2 receptor system in the lower urinary tracts will allow the development of new treatments [5].

Interestingly, ligands for cannabinoid receptors (for both CB1 and CB2), e.g., delta-9-tetrahydrocannabinol (THC) from cannabis, were reported to moderate detrusor stability in the neurogenic bladder population [10]. However, the initial *enthusiasm* shall be tempered by the well-known side effects, i.e., cytotoxicity or addiction [11]. Furthermore, several papers proved that patients suffering from OAB often present with various mental problems, while depression is the most commonly diagnosed one. There is no consensus, however, on whether depression promotes OAB induction or one should perceive OAB as a unique manifestation of psychosomatic disorders [12]. Interestingly, we have found that administration of corticosterone resulted in the induction of depressive and DO symptoms in animals that were restored by the inhibition of corticotropin-releasing receptors [12]. We further proposed cannabinoid ligands as the potential candidates for the treatment of overactive bladder, as the filing phase urodynamic parameters analyzed in the inflamed condition after administration of corticosterone in our model were subsequently normalized due to the administration of cannabinoidergic compounds [13]. Then, N-(2-Chloroethyl)-5Z,8Z,11Z,14Z-eicosatetraenamid (ACEA) is a highly selective CB1R agonist that was found to possess neuroprotective properties [14]. In the recent paper by Jones et al., ACEA ameliorated the hyperalgesic effects and increased bladder contractility in the rat model of acrolein-induced cystitis [15]. The animal model used by the authors revoked similar urodynamic and hyperalgesic findings to those found in patients with interstitial cystitis/bladder pain syndrome (IC/BPS) [15]. Furthermore, antidepressant-like properties of ACEA have also been studied in mice models, revealing the link between psychiatric conditions [16], especially depression and OAB.

ACEA is mainly studied in the neurosciences [14], so we opted for its incorporation in the study on lower urinary tract function in our animal model, as there is growing need to increase the knowledge on the cannabinoid ligands in the treatment of overactive bladder. This is the first study that aimed to determine if the administration of ACEA, a selective cannabinoid CB1 receptor agonist, could reverse the effects of corticosterone (CORT), which induced depressive-like behavior in pre-clinical studies and evoked DO symptoms [12]. Simultaneously, we intended to investigate whether the administration of ACEA with anti-depressive properties impacts the biochemical parameters’ characteristic of bladder overactivity, other than DO cystometric parameters, the locomotor activity of animals, and their behavior in the forced swim test (FST). A rat model of CORT-induced bladder overactivity was adopted for the study, which was developed in our previous experiments [13,17].

## 2. Results

### 2.1. The Effects of ACEA on Corticosterone (CORT)-Induced Changes in Cystometric Parameters

Recently, we confirmed that CORT may induce detrusor overactivity symptoms in animals [12]. Here, we found that ACEA ameliorated CORT-induced changes in cystometric parameters analyzed in both voiding and storage phase (Table 1). The storage phase was characterized by the improvement in the following values: threshold pressure (TP), basal pressure (BP), bladder compliance (BC), volume threshold to elicit NVC (VTNC), detrusor overactivity index (DOI), and finally, non-voiding contraction frequency (FNVC) (Table 1). Then, the voiding parameters that were affected by CORT and ameliorated by ACEA administration were as follows: micturition voiding pressure (MVP), intercontraction interval (ICI), voided volume (VV), post-void residual (PVR), and area under the pressure curve (AUC).

### 2.2. The Effects of ACEA on Corticosterone (CORT)-Induced Behavioral Changes

We failed to reveal any effects of CORT on locomotor activity in the tested animals and, subsequently, no changes were observed after ACEA administration, as well (Table 2). However, CORT prolonged the immobility time in the forced swim test (FST) and, interestingly, those values were lowered by the action of ACEA.

### 2.3. The Effects of ACEA on Corticosterone (CORT)-Induced Changes in the Expression Levels of c-Fos in Central Micturition Areas

**Figure 1:**
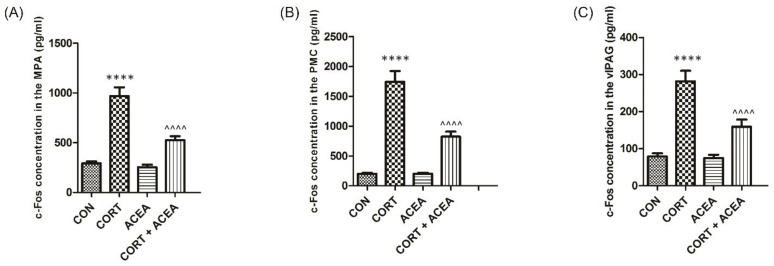
The effects of ACEA (0.3 mg/kg/day i.p., for 7 consecutive days) on c-Fos expressions in the neuronal voiding centers: (**A**) medial preoptic nucleus (MPA), (**B**) pontine micturition center (PMC), and (**C**) ventrolateral periaqueductal gray (vlPAG) after the induction of overactive bladder with corticosterone (CORT). Concentration of c-Fos in each group [pg/mL]: control group (CON), CORT group (CORT), ACEA only group (ACEA) and CORT group treated with ACEA (CORT + ACEA). Each experimental group consisted of 12 animals. Values are expressed as the mean ± S.E.M.**** or ^^^^ *p* < 0.0001. One-way ANOVA: for MPA: F(3.44) = 40, *p* < 0.0001; for PMC: F(3.44) = 53, *p* < 0.0001; for vlPAG: F(3.44) = 28, *p* < 0.0001.

c-Fos expression was considerably altered in all the analyzed areas after 14-day administration of CORT when compared to the controls (Figure 1A–C). ACEA alone did not influence on these values (ACEA group). At the same time, we revealed that the ACEA action was potent in decreasing the c-Fos values after CORT exposure in all the studied micturition compartments (Figure 1, CORT plus ACEA vs. CORT group).

### 2.4. The Effects of ACEA on Corticosterone (CORT)-Induced Changes in Biochemical Analyses of Biomarkers in Urine

**Figure 2:**
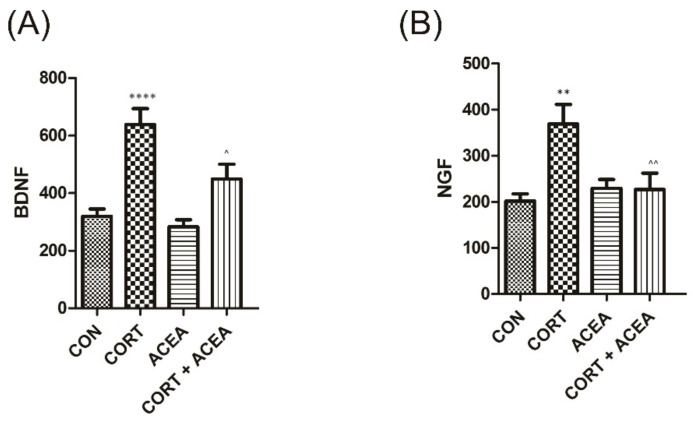
The influence of the 7-day administration of ACEA on biomarkers’ level [pg/mL] in urine: (**A**) brain-derived neurotrophic factor (BDNF), and (**B**) nerve growth factor (NGF) in rats subjected to a 14-day administration of corticosterone (CORT). Each experimental group consisted of 12 animals. Values are expressed as the mean ± S.E.M. **** *p* < 0.0001, ** or ^^ *p* < 0.01., ^ *p* < 0.05. ^ significantly different from the CORT group. One-way ANOVA: for BDNF: F(3.44) = 15, *p* < 0.0001, and for NGF: F(3.44) = 6.3, *p* < 0.01; CON, control.

The administration of ACEA did not result in changes in the analyzed parameters in urine (Figure 2). On the other hand, subcutaneous injection of CORT induced significant changes in both BDNF and NGF values that were successfully restored by ACEA.

### 2.5. The Effects of ACEA on Corticosterone (CORT)-Induced Changes in Biochemical Analyses of Biomarkers in the Bladder Detrusor Muscle

**Figure 3:**
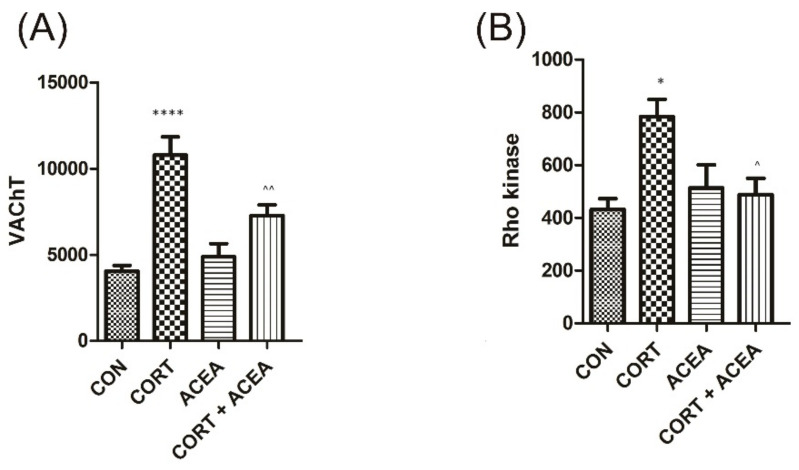
The influence of the 7-day administration of ACEA on biomarkers’ level [pg/mL] in in the bladder detrusor muscle: (**A**) vesicular acetylcholine transporter (VAChT) and (**B**) Rho kinase. Each experimental group consisted of 12 animals. Values are expressed as the mean ± S.E.M. **** *p* < 0.0001, ^^ *p* < 0.01., * or ^ *p* < 0.05 * significantly different from the control group. ^ significantly different from the CORT group. One-way ANOVA: for VAChT: F(3.44) = 17, *p* < 0.0001, and for Rho kinase: F(3.44) = 5.6, *p* < 0.01. CON, control.

The analysis of the biomarkers in the bladder detrusor muscle after administration of CORT revealed a significant increase in both of the tested proteins (Figure 3). ACEA was potent enough to ameliorate the CORT-induced changes on one hand (CORT + ACEA), while it did not affect the values of these particular parameters on the other (ACEA).

### 2.6. The Effects of ACEA on Corticosterone (CORT)-Induced Changes in Biochemical Analyses of Biomarkers in the Bladder Urothelium

**Figure 4:**
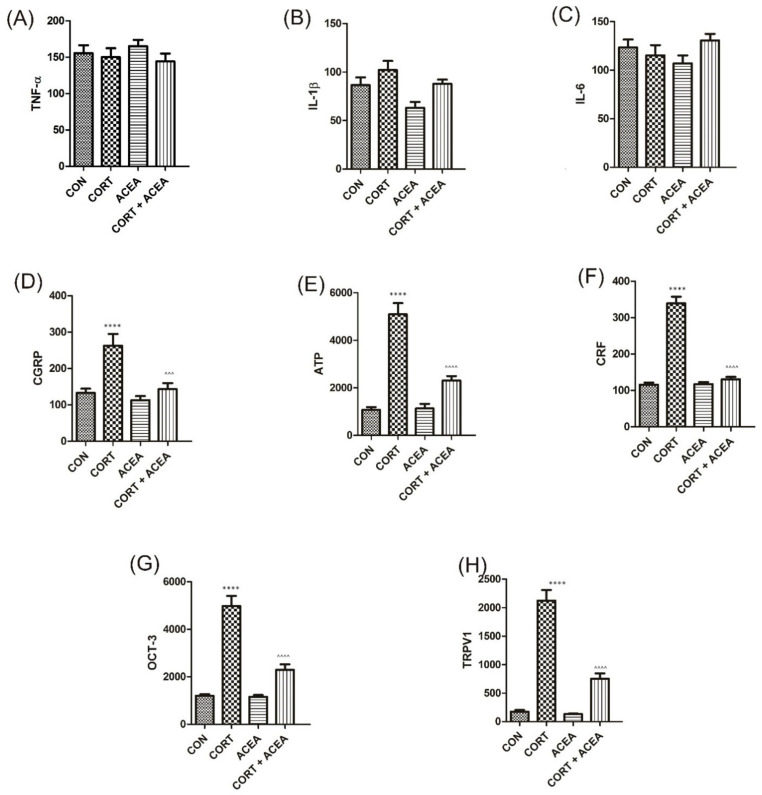
The influence of the 7-day administration of ACEA on biomarkers’ level [pg/mL] in the bladder urothelium: (**A**) tumor necrosis factor α (TNF-α), (**B**) Interleukin 1-β (IL-1β), (**C**) Interleukin-6 (IL-6), (**D**) Calcitonin Gene-Related Peptide (CGRP), (**E**) adenosine triphosphate citrate lyase (ATP), (**F**) corticotropin-releasing factor (CRF), (**G**) Organic Cation Transporter 3 (OCT-3), (**H**) Transient Receptor Potential Cation Channel Subfamily V, Member 1 (TRPV1) in rats subjected to a 14-day administration of corticosterone (CORT). Each experimental group consisted of 12 animals. Values are expressed as the mean ± S.E.M. ^^^ *p* < 0.001, **** or ^^^^ *p* < 0.0001. One-way ANOVA: for IL-1β F(3.44) = 5, *p* < 0.01; for CGRP F(3.44) = 12, *p* < 0.0001; for ATP F(3.44) = 46, *p* < 0.0001; for CRF F(3.44) = 114, *p* < 0.0001; for OCT-3 F(3.44) = 52, *p* < 0.0001; for TRPV1 F(3.44) = 78, *p* < 0.0001. CON control.

No influence on the levels of the analyzed biomarkers in the bladder urothelium after administration of ACEA was noted when compared with the control group (Figure 4). We further observed the effects of CORT on the elevation of levels of several biomarkers (CGRP, ATP, CRF, OCT-3, and TRPV1). Interestingly, no changes were revealed as for pro-inflammatory cytokines (TNF-α, IL-1β, and IL-6) (Figure 4A–C). Finally, ACEA proved to be effective in the reversion of the CORT-induced changes in all the cases (Figure 4D–H).

### 2.7. The Effects of ACEA on Corticosterone (CORT)-Induced Changes in Biochemical Analyses of Biomarkers in Hippocampus

**Figure 5:**
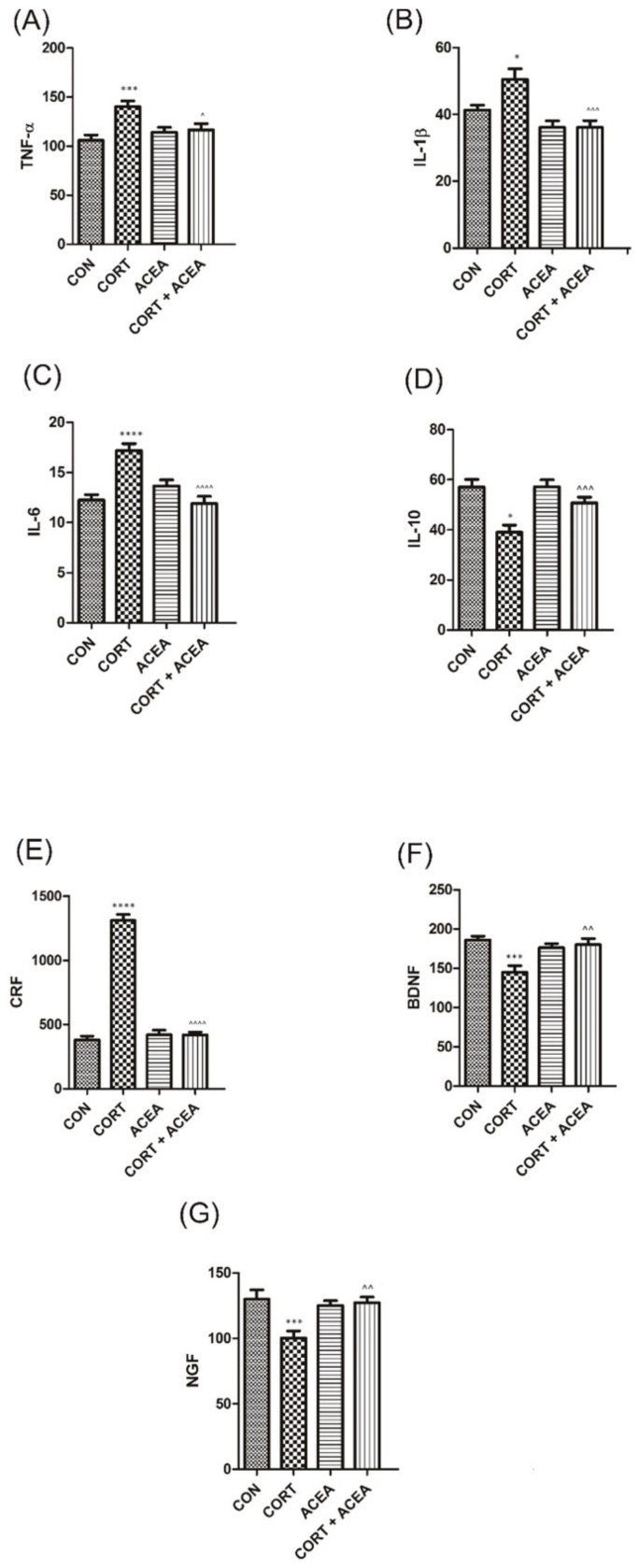
The influence of the 7-day administration of ACEA on biomarkers’ level [pg/mL] in hippocampus: (**A**) tumor necrosis factor α (TNF-α), (**B**) Interleukin 1-β (IL-1β), (**C**) Interleukin-6 (IL-6), (**D**) Interleukin-10 (IL-10), (**E**) corticotropin-releasing factor (CRF), (**F**) brain-derived neurotrophic factor (BDNF), (**G**) nerve growth factor (NGF) in rats subjected to a 14-day administration of corticosterone (CORT). Values are expressed as the mean ± S.E.M. * *p* < 0.05; ^^ *p* < 0.01; *** or ^^^ *p* < 0.001, **** or ^^^^ *p* < 0.0001. * significantly different from the control group, ^ significantly different from the CORT group. One-way ANOVA: for TNF-α F(3.44) = 6.7, *p* < 0.001; for IL-1β F(3.44) = 8.7, *p* < 0.0001; for IL-6 F(3.44) = 14, *p* < 0.0001; for IL-10 F(3.44) = 9.5, *p* < 0.0001; for CRF F(3.44) = 166, *p* < 0.0001; for BDNF F(3.44) = 7.3, *p* < 0.001; for NGF F(3.44) = 7.3, *p* < 0.001. CON control.

Finally, the biomarkers’ levels were analyzed in the hippocampus (Figure 5). We further found the ACEA bared no effect on all the analyzed values in this compartment. On the other hand, CORT significantly increased the values of proinflammatory cytokines (TNF-α, IL-1β, and Il-6) and lowered the levels of IL-10, which acts on the contrary (Figure 5A–D). Furthermore, we observed an increase in CRF and a decrease in BDNF and NGF levels due to CORT injections (Figure 5E–G). Similarly to the actions observed in urine and other analyzed compartments (bladder urothelium and detrusor), we confirmed the efficacy of ACEA to ameliorate CORT-induced changes in the tested parameters (Figure 5A–G).

## 3. Discussion

The rationale for the experimental research in OAB comes from the growing need to develop and exceed the range of medical treatments, keeping in mind well-known side effects of the existing modalities [18]. ACEA, a synthetic agonist of cannabinoid receptor 1, represents a novel concept of incorporation of the cannabinoid-dependent pathway involved in both depressive and OAB symptoms [13,19,20]. Here, we revealed that intraperitoneal administration of ACEA reversed changes in cystometric parameters induced by the 14-day administration of CORT, especially those related to DO. Furthermore, we successfully restored voiding parameters in the analyzed animals affected by CORT. Interestingly, in the paper by Jones et al., the intrathecal root was not successful in reversing acrolein-related urodynamic changes [15]. The authors hypothesized that pain associated with bladder inflammation may be stopped by the induction of spinal CB1R; nevertheless, the experiments revealed that local pathways induced by cystitis were not ameliorated by the action of spinal CB1R [15].

The positive correlation between OAB symptoms and depression is well-known. However, there are controversies regarding OAB as causal factor of affective disorders or the opposite. The third hypothesis states that depression and OAB could simply share common pathological pathways [21]. In such a case, CRF could play a central role, as the blockade of its receptor CRF1 could ameliorate symptoms of depression and OAB [22]. Observed alternations of cytokines and neutrophines in the hippocampus of rats in our study accurately replicated alternations observed in depression. Persistent low-grade inflammation is considered to be a pathophysiological factor for depression. In line with our results, expressions of pro-inflammatory cytokines (IL-1β, TNF-α and IL-6) were increased, whereas the expression of anti-inflammatory cytokines (IL-10 and TGF-β) was decreased in the hippocampus of the previously described rat model of depression [23]. Similarly, the decreased expression of NGF and BDNF in our study was confirmed by clinical and animal studies, in which it has been suggested that depression is associated with the neuronal atrophy caused by low levels of neurotrophins in the hippocampus [24]. CB1 receptors are localized in neuroanatomical structures responsible for depression and their stimulation has been shown to have an antidepressant effect [25]. However, the exact mechanism of action of CB1 stimulation on observed alternation in the central nervous system is not known yet. Finally, an additional aspect of antidepressant-like properties of ACEA was examined in the FST, which represents a behavioral test used in rodents that enables the assessment of the efficacy of such treatment [16]. In the paper by Rutkowska et al., a dose of 2.0 mg/kg ACEA (dosages assessed 0.5–2.0 mg/kg i.p. in BALB/c mice) was proved to be similarly effective when compared with fluoxetine, or even more effective when used concomitantly [16]. Here, we found that the prolongation of the immobility time in the FST in rats induced by CORT was even lower than in the control group due to ACEA administration.

Then, we focused on c-Fos expression in central micturition areas after 14-day administration of CORT, which was elevated in all the analyzed compartments. Interestingly, we observed that ACEA normalized the c-Fos values after the administration of CORT. c-Fos is deemed to be a marker of neuronal activity [26]. Chronically administered CORT is expected to enhance c-Fos expression in some brain areas, and decrease it in others [26]. Observed overexpression of c-Fos in PMC, vlPAG, and MPA after CORT treatment implies increased neuronal activity in central micturition centers and could replicate OAB symptoms in rats [27]. As the link between c-Fos expression in micturition centers and the activation of CB1 receptors by ACEA may be indirect, it has been shown that in depression, the hallmark of which is hypothalamic-pituitary-adrenal (HPA) axis hyperactivation, stimulation of endocannabinoid system through CB1 receptors suppresses the HPA axis [28].

We observed significant changes in the concentrations of the respective biomarkers analyzed after the administration of CORT in urine, the bladder detrusor muscle, the bladder urothelium and the hippocampus, respectively. Firstly, i.p. injection with ACEA resulted in the restoration of values of both BDNF and NGF in urine. Neurotrophins best studied in the context of OAB and depression, i.e., NGF and BDNF, are responsible for sensory afferent nerve plasticity [29]. The overexpression of NGF and BDNF both in the bladder and in the neuronal pathway is a well-established phenomenon in animal models of OAB, as well as in patients with OAB syndrome [30]. To the best of our knowledge, there are no data in the literature that could explain the direct effect of ACEA on NGF expression after CORT stimulation in the bladder and in the urine. On the other hand, CB1 activation is known to interfere with NGF action. Wang et al. have shown in vitro in mice that dorsal root ganglion afferent neurons activation via CB1 receptors inhibited NGF-induced TRPV1 sensitization by suppression of the AKT pathway [20]. Likewise, systematically administered ACEA could have an inhibitory effect on neural circuitry pathways responsible for micturition [20,31].

Further experiments on biomarkers in the bladder detrusor muscle revealed that both VAChT and Rho kinase levels that were elevated due to CORT action were lowered by the influence of ACEA. The RhoA/Rho-kinase signaling pathway appears to be associated with depression, and its expression is influenced by glucocorticosteroids [32]. The RhoA/Rho kinase signaling pathway is also involved in bladder basal tone, and the stimulation of RhoA/Rho kinase may lead to increased involuntary bladder contractions [33]. Additionally, the increased expression of VAChT, which may be induced by BDNF, implies augmented cholinergic transmission [34].

With explanatory intention, the biomarkers of bladder urothelium were analyzed after the co-administration of CORT and ACEA. We found that several substances, including CGRP, ATP, CRF, OCT-3, and TRPV1, were elevated due to CORT, but for pro-inflammatory cytokines (here we analyzed TNF-α, IL-1β, and IL-6). We further observed the potency of ACEA to ameliorate these effects of CORT in all the analyzed cases. CGRP and TRPV-1 were colocalized in afferent unmyelinated C nerve fibers to be found in urothelium and suburothelium [35,36], and are thought to be responsible for phasic activity in the bladder by local axonic reflexes [37]. The density of CGRP immunoreactive fibers is increased in patients with OAB, which suggests their role in the pathophysiology of the disease [38]. Similarly, TRPV1 mRNA expression is increased in patients with sensory OAB [36]. Thus, the increased expression of CGRP and TRPV-1 suggests afferent C fibers activation [39], and may contribute to the observed cystometric dysfunction in our study. Interestingly, it has been shown that CB1 stimulation by ACEA prevented NGF-induced TRPV1 sensitization [40]. OCT3 is responsible for the non-neuronal release of ACh from the urothelium [41]. Decreased expression in both VAChT and OCT3 are the signs of ACEA effectiveness in the reduction in ACh release, which is a mainstay of OAB therapy. ATP is released from the urothelium during bladder distention and activates purinergic receptors P2X, localized on suburothelial afferent nerve fibers [36]. It is responsible for bladder sensation and mediates the voiding reflex [36]. Intravesical infusion of ATP directly causes overactivity and, what is more, purinergic receptors (P2X3 and P2X2/3) are postulated to lower the sensory threshold of afferent C fibers [36]. For the following reasons, ATP could cause detrusor overactivity and is considered a biomarker for OAB [42]. In rat bladder stripes, the activation of CB1 receptor-induced conversion of ATP to cyclic adenosine monophosphate (cAMP), through K_ATP_ channels, leads to calcium channel opening and muscle relaxation [43]. Finally, no changes in pro-inflammatory cytokines were noted in the above-mentioned experiments within bladder urothelium, while cystitis can contribute to the DO per se, and a local inflammation as a pathophysiological factor of OAB has already been excluded [22].

Among possible limitations of our study, it should be emphasized that the properties of ACEA revealed in our experiments cannot be directly extrapolated in the human population, which calls for clinical studies. As for dosages and schedules implemented in the study, they originated from preliminary unpublished data, as no exact information is available in the literature.

## 4. Materials and Methods

All applied procedures were approved by the Local Ethics Committee (4326LKB/323/2022), and they were performed in accordance with binding European law related to the experimental studies on animal models.

### 4.1. Animals

48 female Wistar rats (weighted originally 200–225 g) were included in the study. Animals were placed individually in the metabolic cages (3700M071, Tecniplast, West Chester, PA, USA) in environmentally controlled rooms (as described previously [12,13] with unlimited access to water and food. Rats were randomly cohorted into the four following experimental groups (12 animals per group):

1. Control group receiving vehicle for 14 days plus vehicle for 7 days (the control group, CON);
2. Corticosterone 20 mg/kg/day for 14 days plus vehicle for 7 days (CORT);
3. Vehicle for 14 days plus ACEA for 7 days (ACEA; 0.3 mg/kg/day);
4. Corticosterone 20 mg/kg/day for 14 days plus ACEA for 7 days (CORT + ACEA).

All animals were experimentally naive and tested once. Each experimental group consisted of 12 animals.

### 4.2. Drugs

The following drugs were used:

- Corticosterone (CORT) (Tocris Bioscience): (11β)-11,21-Dihydroxypregn-4-ene-3,20-dione—was given subcutaneously at a daily dose of 20 mg/kg for 14 days as described elsewhere (12),
- ACEA (Tocris Bioscience): N-(2-Chloroethyl)-5Z,8Z,11Z,14Z-eicosatetraenamide—a potent and highly selective CB1 receptor agonist (Ki = 1.4 nM), which displays >1400-fold selectivity over CB2 receptors. ACEA was administered intraperitoneally (i.p.) at a daily dose of 0.3 mg/kg for 7 days. The doses of the administered agents were selected on the basis of the results of our previous experiments and the literature data and were confirmed/adjusted in our laboratory in preliminary non-published experiments. The control animals received volume-matched injection of vehicle.

### 4.3. Surgical Procedures

All the surgical procedures were conducted as described previously, under standardized anesthesia [12,44]. Briefly, anesthetized animals were placed in a supine position and no spontaneous movement and no withdrawal response to noxious stimuli was used as tools to confirm the adequate depth of anesthesia. The surgically prepared abdominal wall was opened through a vertical midline incision. The bladder was gently dissicated from the adjacent organs. A double lumen polyethylene catheter was installed via small incision into the bladder dome and sutured, as described previously [12,13,44]. Then, the catheter was placed subcutaneously and exteriorized in the retroscapular area, where it was connected with a plastic adapter, to minimalize the risk of spontaneous removal. Finally, the abdomen was closed in multiple layers. The animals received 100 mg of cefazolin sodium hydrate (Biofazolin; Sandoz) for the prevention of urinary tract infection.

### 4.4. Conscious Cystometry

Cystometric studies were performed in conscious unrestrained rats 3 days after the last injection of ACEA, as described previously [17,45]. Conscious cystometry was conducted by slowly filling the bladder with physiological saline (stable rate of 0.05 mL/min, i.e., 3 mL/h, room temperature) to induce repetitive voiding. The analogue signal obtained from the pressure transducer was converted using the Polyview system (Grass Instruments). Micturition volumes were analyzed using a fluid collector attached to a force displacement transducer (FT03C; Grass Instruments). Both transducers were connected to a polygraph (7 DAG; Grass Instruments). Cystometry profiles and micturition volumes were recorded continuously on a Grass polygraph (Model 7E; Grass Instruments) and presented graphically. The data were analyzed using a sampling rate of 10 samples/s. The measurements in each rat represent the average of five bladder micturition cycles after obtaining repetitive voiding. All procedures were blinded as they were performed by a person unaware about the treatments.

### 4.5. Forced Swim Test

A forced swim test was carried out according to the method of Porsolt et al. [46]. Briefly, the animals were placed individually in 48-cm height glass cylinders filled with water at 23–25 °C. Rats returned to their home cages after 15 min in water. 24 h after the forced swim, rats were retested for 5 min under identical conditions. Retests were recorded from the side of the cylinders and scored using a behavioral sampling method by the blinded person. The rat was ranked as immobile when it remained floating passively, performing slow-motion movements to keep its head above the water.

### 4.6. Locomotor Activity

A Digiscan apparatus was used for the purpose of the locomotor activity assessment, as described previously [17]. Briefly, it monitored animal locomotor activity via a grid of invisible infrared light beams. The body of the animal placed in the Digiscan interrupted these beams showing its position, and the interruption of any beam was assessed as an activity score. Cumulative counts were compiled and downloaded every 15 min into the OMNIPRO data collection program. A 15-min habituation period was implemented before experiments. Horizontal activity was assessed that was defined as the total number of beam interruptions that occurred in the horizontal sensor during 1 h of measurement. All procedures were performed by a person unaware about the treatments.

### 4.7. Biochemical Analyses

In the biochemical analyses, the following biomarkers were analyzed. C-Fos (c-Fos; MyBioSource, MBS729725) expression was measured in the central micturition areas: medial preoptic area (MPA), the ventrolateral periaqueductal gray (vlPAG), and pontine micturition center (PMC) (as described in detail in Section 4.7.1 below). The levels of Vesicular acetylcholine transporter (VAChT; LifeSpan BioSciences, CN LS-F12924-1) and Rho Kinase (ROCK1; LifeSpan BioSciences, LS-F32208) were assessed in bladder detrusor muscle, while the concentrations of nerve growth factor (NGF; LifeSpan BioSciences, CN LS-F25946-1) and brain-derived neurotrophic factor (BDNF; PROMEGA, CN G7610) were analyzed in urine. As for the bladder urothelium, the following markers were measured: tumor necrosis factor α (TNF- α, LifeSpan BioSciences; LS-F5193), interleukin 1β (IL-1β, Cloud-Clone; SEA563Ra), interleukin 6 (IL-6, LifeSpan BioSciences; LS-F25921-1), Calcitonin Gene-Related Peptide (CGRP; Biomatik, CN EKU02858), ATP Citrate Lyase (ATP; LifeSpan BioSciences, LS-F10730), corticotropin-releasing factor (CRF; Alpco, Salem, NH, USA, CN 48-CRFMS-E01), Organic Cation Transporter 3 (OCT3; antibodies-online, CN ABIN6227163), and Transient Receptor Potential Cation Channel Subfamily V, Member 1 (TRPV1; LSBio, LS-F36019). Finally, the respective values of TNF-α, IL-1β, IL-6, interleukin 10 (IL-10, LifeSpan BioSciences; LS-F5081), CRF, BDNF, and NGF were measured in the hippocampus (as described in Section 4.7.2 below). All measurements were carried out according to the manufacturers’ instructions. Each sample was measured in duplicate. The results are presented in pg/mL.

#### 4.7.1. Determining the Expression Levels of c-Fos in the Central Micturition Areas

We implemented the stereotactic atlas of the rat’s brain and the bregma [47] to isolate the PMC, vlPAG, and MPA, as described by Kim et al. [48]. Ten sections on average per region were obtained from each animal. The ELISA test was used for the measurement of c-Fos levels in the supernatants, as described in the above Section 4.7.

#### 4.7.2. Determining the Expression Levels of Biomarkers in the Hippocampus

Based on the stereotactic atlas of the rat’s brain and the bregma [47], the hippocampus was isolated and homogenized as described elsewhere [48,49]. Using commercially available ELISA tests, the concentrations of the respective biomarkers in the hippocampus supernatants were analyzed according to the manufacturer’s manual.

### 4.8. Study Design

Three days after the last injection of ACEA, the following studies were carried out: cystometry, the Porsolt test, and locomotor activity measurement. After the cystometric and behavioral studies, the animals were killed by decapitation and their brains and urinary bladder tissue were collected.

### 4.9. Statistical Analysis

The statistical analyses were based on two-way analysis of variance (ANOVA) followed by Bonferroni’s post hoc test. The results from the experiments described above were presented as the means ± standard error of the mean (SEM). The differences between the tested group were regarded as statistically significant when *p* < 0.05.

## 5. Conclusions

In the light of the discussed associations, further studies on the cannabinoid pathways and possible interactions involved in the pathology of OAB are needed. However, ACEA was found to be effective in our model of OAB and depression induced by CORT administration, which, in turn, represents an example of the existing link between OAB and depression via cannabinoid receptor ligands.

## Figures and Tables

**Table 1:** The effects of ACEA on corticosterone (CORT)-induced changes in cystometric parameters.

	Control	Corticosterone	ACEA	Corticosterone + ACEA
***Storage phase***				
**Threshold Pressure** **(TP, cm H_2_O)**	8.9 ± 1	6.7 ± 1.2 **	9.2 ± 1.7 ^^^	8.5 ± 1.8 ^
**Basal Pressure (BP, cm H_2_O)**	3.6 ± 0.49	5.1 ± 0.91 ****	2.9 ± 0.69 ^^^^	3.1 ± 0.84 ^^^^
**Bladder Compliance (BC, mL/cm H_2_O)**	0.31 ± 0.050	0.24 ± 0.053 **	0.28 ± 0.024	0.34 ± 0.051 ^^^^
**Volume Threshold to Elicit NVC (VTNC, %)**	75 ± 7.5	55 ± 8.2 ****	67 ± 10 ^	67 ± 8.3 ^^
**Detrusor Overactivity Index (DOI, cm H_2_O/mL)**	30 ± 15	851 ± 291 ****	27 ± 12 ^^^^	206 ± 56 ^^^^
**Non-voiding Contractions Frequency (FNVC, times/filling phase)**	0.36 ± 0.24	7.3 ± 1.2 ****	0.3 ± 0.17 ^^^^	3.8 ± 0.97 ****^^^^
**Non-voiding Contractions Amplitude (ANVC, cm H_2_O)**	2.9 ± 0.49	3.5 ± 0.91	2.5 ± 0.39	3.4 ± 0.64
*Voiding phase*				
**Micturition Voiding Pressure (MVP, cm H_2_O)**	49 ± 4.9	38 ± 7 **	52 ± 8 ^^^	41 ± 9 ^
**Intercontraction Interval (ICI, s)**	1049 ± 105	863 ± 107 ***	1085 ± 116	990 ± 124 ^
**Voided Volume (VV, mL)**	0.97 ± 0.13	0.73 ± 0.081 ***	0.94 ± 0.13 ^^	1 ± 0.18 ^^^^
**Post-void Residual (PVR, mL)**	0.071 ± 0.011	0.093 ± 0.015 **	0.074 ± 0.018 ^	0.073 ± 0.016 ^
**Area Under the pressure Curve (** **AUC, cm H_2_O/s)**	20 ± 2.9	31 ± 3.7 ****	18 ± 2.4 ^^^^	22 ± 2.6 ^^^^

Values are expressed as the mean ± S.E.M. ^ *p* < 0.05; ** or ^^ *p* < 0.01; *** or ^^^ *p* < 0.001, **** or ^^^^ *p* < 0.0001. ^ significantly different from the CORT group. One-way ANOVA: for TP F(3.44) = 6.8, *p* < 0.001; for BP F(3.44) = 21, *p* < 0.0001; for BC F(3.44) = 10, *p* < 0.0001; for VTNVC F(3.44) = 10, *p* < 0.0001; for DOI F(3.44) = 83, *p* < 0.0001; for FNVC F(3.44) = 20, *p* < 0.0001; for ANVC F(3.44) = 6.2, *p* < 0.01; for MVP F(3.44) = 9.5, *p* < 0.0001; for ICI F(3.44) = 8.8, *p* < 0.0001; for VV F(3.44) = 12, *p* < 0.0001; for PVR F(3.44) = 5.4, *p* < 0.01; and for AUC F(3.44) = 45, *p* < 0.0001. TP: threshold pressure (cm H_2_O), BP: basal pressure (cm H_2_O), BC: bladder compliance (mL/cm H_2_O), VTNVC: volume threshold to elicit NVC (%), DOI: detrusor overactivity index (cm H_2_O/mL), FNVC: non-voiding contractions frequency (times/filling phase), ANVC: non-voiding contractions amplitude (cm H_2_O), MVP: micturition voiding pressure (cm H_2_O), ICI: intercontraction interval (s), VV: voided volume (mL), PVR: post-void residual (mL), and AUC: the area under the pressure curve (cm H_2_O/s).

**Table 2:** The influence of ACEA on the immobility time and locomotor activity in rats.

	Control	Corticosterone	ACEA	Corticosterone + ACEA
**Number of movements during 1 h**	6009 ± 1379	5935 ± 1233	5567 ± 1145	6418 ± 1207
**Immobility time (s) (forced swim test, FST)**	183 ± 12	227 ± 20 ****	172 ± 12 ^^^^	169 ± 16 ^^^^

Values are expressed as the mean ± S.E.M. **** or ^^^^ *p* < 0.0001. One-way ANOVA: for forced swim test F(3.44) = 36, *p* < 0.0001.

## Data Availability

The data presented in this study are available on request from the corresponding authors.

## Abbreviations

ACEA	N-(2-Chloroethyl)-5Z:8Z:11Z,14Z-eicosatetraenamid
ANVC	non-voiding contractions amplitude (cm H_2_O);
ATP	adenosine triphosphate citrate lyase (pg/mL);
AUC	the area under the pressure curve (cm H_2_O/s);
BC	bladder compliance (mL/cm H_2_O);
BDNF	brain-derived neurotrophic factor (pg/mL);
BP	basal pressure (cm H_2_O);
c-Fos	AP-1 transcription factor subunit (pg/mL);
CGRP	Calcitonin Gene-Related Peptide (pg/mL);
CRF	corticotropin-releasing factor (pg/mL);
DO	detrusor overactivity;
DOI	detrusor overactivity index (cm H_2_O/mL);
FNVC	non-voiding contractions frequency (times/filling phase);
ICI	intercontraction interval (s);
IL-1β	Interleukin 1-β (pg/mL);
IL-6	Interleukin-6 (pg/mL);
IL-10	Interleukin-10 (pg/mL);
MPA	medial preoptic area;
MVP	micturition voiding pressure (cm H_2_O);
NGF	nerve growth factor (pg/mL);
OAB	overactive bladder syndrome;
OCT3	Organic Cation Transporter 3 (pg/mL);
PMC	pontine micturition center;
PVR	post-void residual (mL);
ROCK	rho kinase (pg/mL);
TNF-	tumor necrosis factor alpha (pg/mL);
TP	threshold pressure (cm H_2_O);
TRPV1	Transient Receptor Potential Cation Channel Subfamily V, Member 1
VAChT	vesicular acetylcholine transporter (pg/mL);
vlPAG	ventrolateral periaqueductal gray;
VTNVC	volume threshold to elicit NVC (%);
VV	voided volume (mL).
